# Mining microbial metatranscriptomes for expression of antibiotic resistance genes under natural conditions

**DOI:** 10.1038/srep11981

**Published:** 2015-07-08

**Authors:** Dennis Versluis, Marco Maria D’Andrea, Javier Ramiro Garcia, Milkha M. Leimena, Floor Hugenholtz, Jing Zhang, Başak Öztürk, Lotta Nylund, Detmer Sipkema, Willem van Schaik, Willem M. de Vos, Michiel Kleerebezem, Hauke Smidt, Mark W.J. van Passel

**Affiliations:** 1 Laboratory of Microbiology, Wageningen University, Wageningen, The Netherlands; 2 Department of Medical Biotechnologies, University of Siena, Italy; 3 Top Institute Food and Nutrition, Wageningen, The Netherlands; 4 Netherlands Consortium for Systems Biology, University of Amsterdam, The Netherlands; 5 Functional Foods Forum, University of Turku, Turku, Finland; 6 Department of Medical Microbiology, University Medical Center Utrecht, Utrecht, The Netherlands; 7 Department of Veterinary Biosciences, University of Helsinki, Finland; 8 Department of Bacteriology and Immunology, Haartman Institute, University of Helsinki, Finland; 9 NIZO Food Research B.V., Ede, The Netherlands; 10 Host-Microbe Interactomics Group, Wageningen University, Wageningen, The Netherlands; 11 National Institute for Public Health and the Environment, Bilthoven, The Netherlands

## Abstract

Antibiotic resistance genes are found in a broad range of ecological niches associated with complex microbiota. Here we investigated if resistance genes are not only present, but also transcribed under natural conditions. Furthermore, we examined the potential for antibiotic production by assessing the expression of associated secondary metabolite biosynthesis gene clusters. Metatranscriptome datasets from intestinal microbiota of four human adults, one human infant, 15 mice and six pigs, of which only the latter have received antibiotics prior to the study, as well as from sea bacterioplankton, a marine sponge, forest soil and sub-seafloor sediment, were investigated. We found that resistance genes are expressed in all studied ecological niches, albeit with niche-specific differences in relative expression levels and diversity of transcripts. For example, in mice and human infant microbiota predominantly tetracycline resistance genes were expressed while in human adult microbiota the spectrum of expressed genes was more diverse, and also included β-lactam, aminoglycoside and macrolide resistance genes. Resistance gene expression could result from the presence of natural antibiotics in the environment, although we could not link it to expression of corresponding secondary metabolites biosynthesis clusters. Alternatively, resistance gene expression could be constitutive, or these genes serve alternative roles besides antibiotic resistance.

Antibiotic resistance genes are ubiquitous in bacterial communities in various ecological niches^1^. Metagenomic studies have revealed the presence of resistance determinants in environments as different as soil, gut, seawater, caves and glaciers^2^,^3^,^4^,^5^,^6^,^7^,^8^. Bacterial communities in these and other ecological niches contain particular subsets of antibiotic resistance determinants, and can thus be seen as natural reservoirs from where they can theoretically spread into other environments and previously non-resistant microbial populations, including pathogens, in a process called horizontal gene transfer^9^,^10^,^11^,^12^. The advantage that resistance genes confer to microorganisms exposed to anthropogenic antibiotic pressure is undisputed. However, the role of resistance genes in bacteria in environments not directly affected by application of antibiotics is less clear^13^, and is not limited to antimicrobial defence. Bacteria can possess proteins that provide antibiotic resistance beyond their primary function^14^. For instance, *Providencia stuartii* possesses an enzyme involved in the acetylation of peptidoglycan, and the similarity of its substrate to certain aminoglycosides causes this enzyme to also provide antibiotic resistance^15^.

Despite a growing body of knowledge about the distribution of antibiotic resistance genes in natural environments (i.e. those environments in which the human impact is limited), little is known about their expression, even though gene expression is a better proxy for functional activity than gene content^16^. We therefore hypothesized that a comprehensive metatranscriptome analysis could provide additional knowledge about resistance gene functioning and regulation. Due to the technical difficulties associated with processing of RNA samples, few metatranscriptome studies of microbial communities have been published^17^,^18^,^19^. However, recent advances, particularly in the field of next generation sequencing technologies, have resulted in increased availability of large metatranscriptome datasets (>10^6^ reads). In order to investigate the actual expression of resistance genes in natural environments, we evaluated both in-house generated as well as public metatranscriptomes. We investigated the expression levels and richness of genes conferring resistance against ten types of antibiotics, and furthermore assessed local production of these antibiotics by investigating the expression of relevant secondary metabolite gene clusters.

## Results

### Cumulative relative expression of antibiotic resistance genes

**Figure 1:**
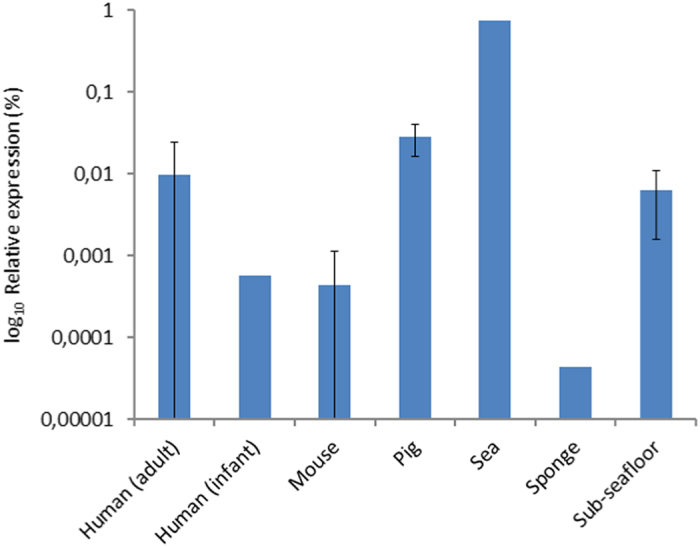
Relative cumulative expression of antibiotic resistance genes in different ecological niches. The relative expression is based on the total of number of reads that aligned to genes of the RED-DB, and calculated as a percentage of the non-ribosomal RNA reads. Error bars represent one standard deviation.

Reads from metatranscriptome datasets from seven different ecological niches (human, mouse and pig intestinal microbiota, sea bacterioplankton, a marine sponge, forest soil and sub-seafloor sediment) were compared with genes from the resistance determinants database (RED-DB, www.fibim.unisi.it/REDDB) using megaBLAST. Per niche, a cumulative total was obtained for reads mapping to the different resistance genes, with the requirement that ≥3 reads aligned per gene. In order to allow for comparison of gene expression in different datasets, we determined the relative abundance of resistance gene transcripts as a percentage of the total number of non-ribosomal RNA reads. Expression of resistance genes was detected in all seven ecological niches (Fig. 1), of which only the pig microbiota was knowingly exposed to antibiotics (i.e. a mixture of neomycin and procaine benzylpenicilline) approximately two to four weeks prior to taking the samples used for RNA extraction. Highest relative expression levels were observed in the sea bacterioplankton sample (0.7%), whereas resistance gene transcripts comprised less than 0.06% of the reads in the other investigated niches. For human adults and pigs relative abundance of resistance gene transcripts was in the same order of magnitude (0.009% ± 0.014 and 0.028% ± 0.012 [s.d.], respectively), whereas it was more than 15-fold lower in mice (4.4 × 10^−4^% ± 6.8 × 10^−4^) and the human infant (5.7 × 10^−4^%).

### Expression of genes conferring resistance against ten types of antibiotics

**Figure 2:**
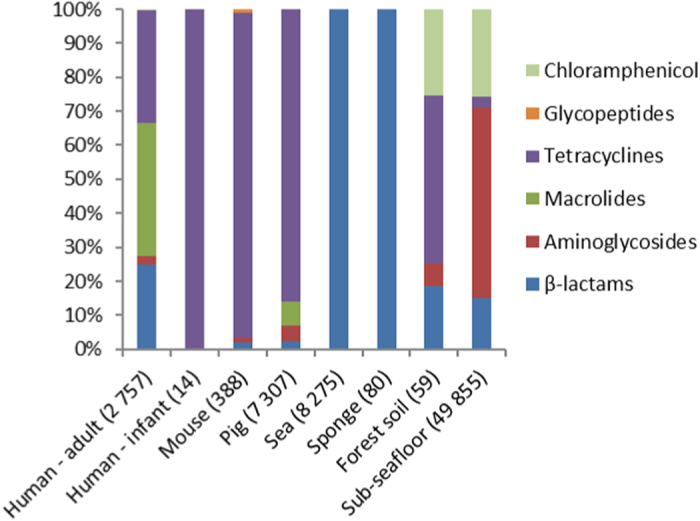
Resistance gene expression profiles for different ecological niches investigated for resistance against ten types of antibiotics. Genes conferring resistance against quinolone, oxazolidinone and trimethoprim antibiotics were not detected. The numbers in parentheses correspond to the total number of reads that aligned to resistance genes.

**Figure 3:**
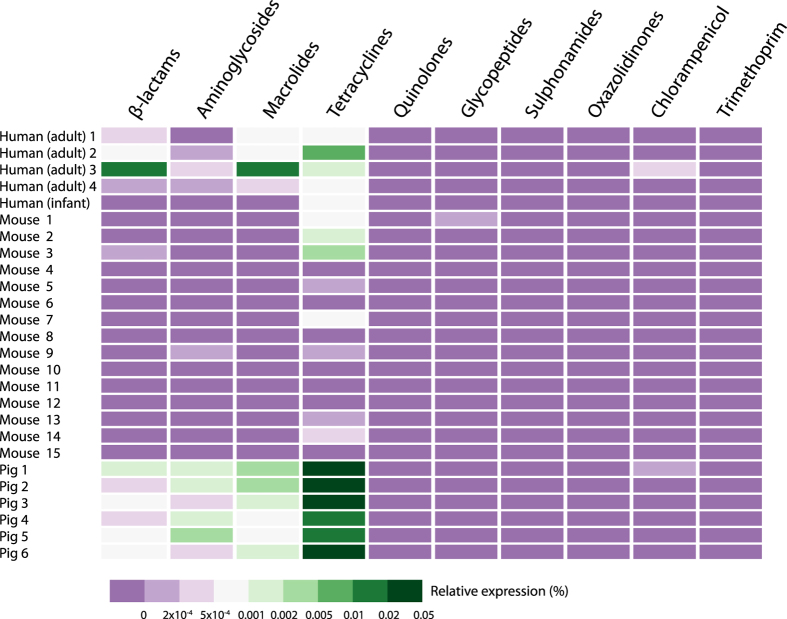
Heat map representing relative expression levels of resistance genes in the gut microbiota of individual humans, pigs and mice. Resistance genes are grouped based on resistance against ten types of antibiotics.

The distribution of reads aligning to resistance genes that confer resistance against ten different types of antibiotics was analysed. Genes in the RED-DB are grouped according to resistance against the following types of antibiotics: aminoglycosides, β-lactams, chloramphenicol, glycopeptides, macrolides, oxazolidinones, quinolones, sulphonamides, tetracyclines and trimethoprim. Expression of genes involved in resistance against most types of antibiotics (6/10) was detected at least in one of the datasets analysed, with the exception of genes conferring resistance against quinolone, oxazolidinone, sulphonamide and trimethoprim antibiotics (Fig. 2). In human gut microbiota β-lactam, aminoglycoside, macrolide and tetracycline resistance genes were expressed. In mice intestinal content samples, in which the cumulative relative expression of resistance genes was the lowest for all gut-associated niches examined here, primarily tetracycline resistance genes were found to be expressed. One-way ANOSIM showed that resistance profiles were not significantly different between different mammalian hosts when comparing the groups of human adults, mice and pigs (global R = 0,089 p = 0.197). A comparison of antibiotic resistance gene expression profiles of the microbiota of individual human adults, pigs and mice indicated there are substantial inter-individual differences within the groups (Fig. 3). In the intestinal samples obtained from the human infant, only the expression of a tetracycline resistance gene was detected. The profiles of expressed resistance genes observed in forest soil and sub-seafloor sediment communities stood out because expression of chloramphenicol resistance genes made up ≥20% of the cumulative total whereas in the other ecological niches they were not detected. In sea bacterioplankton and the marine sponge the expression of two β-lactam resistance genes was detected, namely *blaTEM-1* and *blaTEM-116*. These genes share 97% nucleotide identity and therefore it is possible that in reality only a single β-lactam resistance gene is being expressed that is closely related to the two known genes. In sea bacterioplankton as much as 0.7% of the non-ribosomal transcripts aligned with the two *blaTEM* genes.

### Richness of expressed antibiotic resistance genes

**Figure 4:**
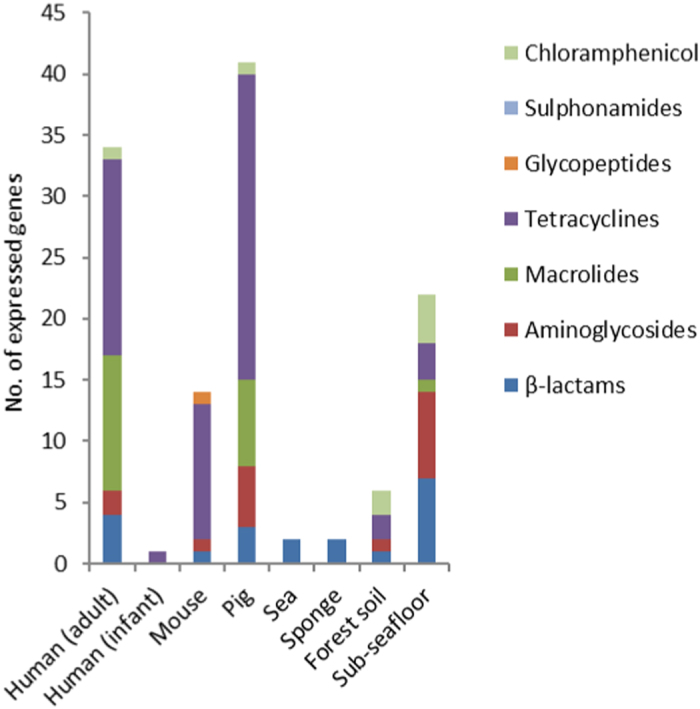
Richness of expressed resistance genes detected in the different ecological niches, based on the number of unique genes that were detected while using RED-DB entries clustered according to a 97% identity threshold.

Read mapping was performed against single gene representatives of gene clusters defined by a 97% sequence identity threshold, in the following referred to as unique genes, and as a result, the number of detected genes can be taken as a measure for resistance gene richness. In human adult and pig microbiota the highest numbers of unique resistance genes were detected, namely 34 and 41 respectively. In the sea bacterioplankton and sponge metatranscriptomes, only two different resistance genes were detected. Detected resistance genes were classified according to which type of antibiotics they confer resistance (Fig. 4, Supplementary Table S2). It should be noted that the number of unique genes detected per niche is influenced by dataset size, sampling date, and the number of sampling sites from which data was analysed. Rarefaction curves in most cases (19/29) did not reach an asymptote, which suggests that resistance gene richness is underestimated to some extent (Supplementary Fig. S1). Regression analysis was performed to further investigate this feature for the human and pig microbiota, as for these only the rarefaction curve of human adult individual 1 reached saturation (Supplementary Table S1), and because the highest number of unique resistance genes was detected in these niches. Regression analysis equation two provided the best fit, of which the results indicate that in most cases (8/10) the detected number of resistance genes is less than one gene different with respect to the expected number of resistance genes at infinite sample size. Therefore the datasets are expected to be sufficiently large to detect most of the resistance genes that were expressed.

### Expression of secondary metabolite biosynthesis (SMB) clusters

**Figure 5:**
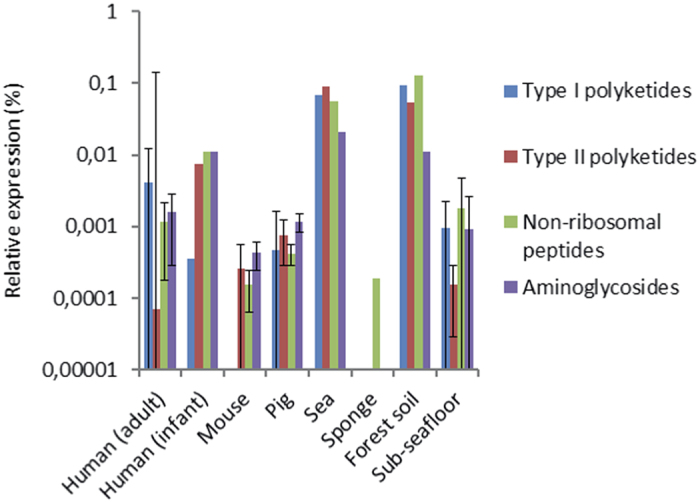
Cumulative relative expression of secondary metabolite biosynthesis domains involved in the production of type I polyketides, type II polyketides, non-ribosomal peptides and aminoglycosides. Error bars represent one standard deviation.

We also investigated whether the expression of antibiotic resistance genes can be associated with local antibiotic biosynthesis. To this end, we used the antibiotics and secondary metabolite analysis shell (antiSMASH) pipeline, which focuses on the production of secondary metabolites, amongst which are also antibiotics^20^. Profile hidden Markov Models (hMMs) from antiSMASH of domains of enzymes involved in the construction of the backbones of various types of antibiotics were used to investigate antibiotic biosynthesis. We investigated genes responsible for the biosynthesis of type I and II polyketides, non-ribosomal peptides and aminoglycosides/aminocyclitols (see Supplementary Table S3 for the domains and the complete results), which can be involved in, respectively, the biosynthesis of macrolides, tetracyclines, β-lactams and aminoglycosides (Fig. 5). In the gut microbiota of humans (adult and infant), pigs and mice, the marine sponge and sub-seafloor sediment, very little expression of SMB genes was detected. The lowest relative expression of SMB genes was found in human individual one, for whom only a single domain was detected by just five reads, suggesting that expression of SMB genes was very low. The expression of SMB genes in sea bacterioplankton and forest soil was at least an order of magnitude higher than in the other investigated environments. No associations between the expression of specific SMB clusters and resistance genes were uncovered, and their cumulative expression levels varied per ecological niche (Supplementary Fig. S2).

## Discussion

Our explorative metatranscriptome analysis showed that antibiotic resistance genes are not only present, but also expressed in a broad variety of natural environments, exemplified here by samples derived from the intestinal tract of humans, mice, as well as from sea bacterioplankton, a marine sponge, forest soil and sub-seafloor sediment. Differences with respect to resistance gene expression profiles were observed between the ecological niches, and resistance gene richness was highest in human and pig microbiota. We find within-niche variability in resistance gene expression in gut microbiota of individual human adults, pigs and mice, which is consistent with metagenome-level studies wherein inter-individual variation in resistance potential was observed^21^,^22^. It should be noted that the resistance profiles observed in this study are not necessarily representative for specific habitats since even samples from the same habitat but taken at different locations have been known to differ more than 100-fold in terms of relative expression levels of individual resistance genes^23^. On the other hand, microbial resistomes have been shown to cluster on habitat when soil, human and water resistomes were compared^24^. Expression of resistance genes against tetracyclines and β-lactams was most abundant in the gut microbiota of mammals and bacterial communities residing in aquatic environments, respectively. In the human gut microbiota specifically, resistance genes against β-lactams, macrolides and tetracyclines cumulatively comprised >90% of the total expression. Resistance genes against these types of antibiotics were also most abundant in metagenome datasets of human gut microbiota^21^. We did not detect the expression of resistance genes against quinolone, oxazolidinone, sulphonamide and trimethoprim antibiotics. These antibiotics are all synthetic, and therefore these results might indicate that there has not yet been sufficient time for the corresponding resistance genes to spread into environmental reservoirs^4^. In the pig microbiota β-lactam and aminoglycoside resistance genes were detected, which might have resulted from these animals having received neomycin and procaine benzylpenicilline two to four weeks prior to sampling.

The expression of polyketide type I and II, non-ribosomal peptide, and aminoglycoside/aminocyclitol biosynthesis genes, which can potentially be involved in antibiotic biosynthesis, was detected in forest soil and sea bacterioplankton. This is consistent with previous reports^25^,^26^,^27^. In turn, in gut microbiota there was very little indication for potential antibiotic biosynthesis. Therefore, since antibiotic pressure seems to be absent in the sampled gut microbiota, it can be argued that antibiotic resistance gene expression in gut microbiota is either constitutive or controlled at least in part independently of the presence of antibiotics. Past studies already indicated that that expression of antibiotic resistance genes can be increased following mutations or movement of genetic elements^28^. As such, under antibiotic pressure, resistance genes that are constitutively expressed at low levels could become more active. For example, the expression of the *ampC* gene, which was detected in all human adults, has been shown to vary depending on a single mutation in the gene promoter^29^.

There are two limitations to the undertaken approach to detect the expression of resistance genes that warrant consideration. The first limitation is that the number of characterized and deposited resistance genes in the RED-DB differs per ecological niche. As a consequence the number of expressed resistance genes can be underestimated if habitats have been studied less extensively. The second limitation is that we require the detection of 200 bp sequences instead of full-length resistance genes, and that as a result genes might be detected with other functional roles that share homology with resistance genes. Still, the cut-offs we used were conservative compared to those applied in previous studies^3^,^30^.

In human adult gut microbiota a large fraction of expressed resistance genes confer resistance to macrolide antibiotics. For this reason, and also since there is no indication of macrolide biosynthesis (in three out of four of the human adults), it can be suggested that the expression of macrolide resistance genes is either a result of prior anthropogenic antibiotic pressure as a result of which these genes are still constitutively expressed at a low level, or that the corresponding proteins serve other roles besides antibiotic resistance. A number of alternative physiological and metabolic functions have been proposed for two genes in *M. tuberculosis* that confer resistance to aminoglycosides, such as immune modulation and alleviation of cellular stress^31^. Nevertheless, the general consensus in literature is that the presence of antibiotic resistance genes in the gut microbiota is the result of anthropogenic antibiotic use, and there are numerous studies to support this claim^22^,^32^,^33^. In contrary to what was observed in humans and pigs in this study, in mice gut microbiota no macrolide resistance genes were found to be expressed, a result that could well be explained by lower exposure of mice to macrolide antibiotics, or antibiotics in general. This might also explain the overall lower levels and diversity of resistance gene expression detected in the gut microbiota of these animals. In the gut microbiota of the human infant expression of two tetracycline resistance genes was detected, as opposed to a variety of resistance genes in human adults. This could be because intestinal microbiota of babies generally have lower species diversity^32^ and therefore species that contain resistance genes have not colonized the gut at levels that would allow detection of their transcripts. Remarkably, the human infant had not been exposed to antibiotics prior to the sampling and hence the observed naïve expression of tetracycline resistance genes indicates that antibiotics are not needed to trigger expression of cognate resistance genes. Indeed, one of the two transcribed tetracycline resistance genes shows high identity with the *tetM* gene which is known to be constitutively expressed but highly mobile as found in various conjugative transposons, such as Tn*916*^33^,^34^.

Surprisingly, in sea bacterioplankton, the expression of AR genes was more than one order of magnitude higher than in any other ecological niche. It was comprised of two highly similar β-lactamase genes, namely *blaTEM-1* and *blaTEM-116*. The same two genes were also found to be expressed in a metatranscriptome dataset from the marine sponge *Crambe crambe*. The *blaTEM-1* gene is commonly present in cloning vectors, and therefore lab contamination can be suspected, however, this possibility is disputed by the fact that the gene is detected in two separate marine metatranscriptome datasets that have been independently generated. The functional role of a β-lactamase in a marine environment is yet unknown. A substrate for this enzyme might be periplasmic or cell-wall bound, as excreted β-lactams could quickly be diluted to negligible concentrations in the open sea. On the other hand, the β-lactamase could also have an extra-cellular role if the bacteria occasionally reside in places with a high cell density (micro niches), such as corals or sponges. Roles of these resistance genes in (the disruption of) cell signalling and antimicrobial activity can be hypothesized^13^. Another possibility is that these β-lactamases have no role in the aquatic environment but that they appeared through resistance gene dissemination, such as via human-derived sewage or animal faeces^35^.

Acquired resistance to antibiotics is often associated with a fitness cost, which translates itself into a reduced bacterial growth rate. It has therefore been proposed that in an antibiotic-naïve environment in the long term, resistant strains will be outcompeted by susceptible strains^36^,^37^. Still, Andersson and Hughes (2010) argued that reversal to a susceptible state proceeds so slowly, if at all, that the fitness cost has no practical importance. Moreover, it cannot be excluded that resistance genes co-localize, i.e. they are genetically linked, or that compensatory mutations have occurred that abolish fitness costs. It has been shown that selection for resistant bacteria occurs at antibiotic concentrations far below the minimal inhibitory concentration^38^. This suggests that resistance can be maintained in bacterial populations even if antibiotics are produced locally at a low concentration, or as a result of anthropogenic intervention.

In summary, a broad diversity of resistance genes was found expressed under natural conditions in the gut microbiota of mammals, as well as in aquatic and terrestrial bacterial communities, with niche-specific differences in relative expression levels and diversity of transcripts. Although the expression of antibiotic resistance genes could not be associated with expression of relevant secondary metabolite biosynthesis clusters, we do see a rich functional reservoir of resistance genes that could be accessible by human pathogens.

## Methods

### Metatranscriptome data

In addition to in-house generated metatranscriptome datasets, literature was searched for available metatranscriptome datasets that contain at least 1 × 10^6^ reads (see Supplementary Table S4 for all datasets included in this study and their accession numbers). The analysed metatranscriptome data were generated from the intestinal microbiota of four human adults, one human infant, six pigs and 15 mice, as well as from the orange-red encrusting sponge (*Crambe crambe*), forest soil^39^, sub-seafloor sediment^40^ and sea bacterioplankton^41^. Except for the pig microbiota, none of the environments that were sampled did experience anthropogenic antibiotic pressure. The pig microbiota metatranscriptome data were included because pigs regularly receive antibiotics during intensive farming, and as such expression of resistance genes in this niche is of particular interest^42^. The human adult metatranscriptome data were obtained from ileostoma effluent samples of 4 human adults^43^, as described by Leimena *et al.*^44^. The metatranscriptome data of the human infant was generated from faecal samples collected from a Finnish baby girl at three time points in the first six months of life (at the ages of 131 [sample 2B], 165 [samples 2D_1 and 2D_2] and 171 [sample 2E] days). Multiple time points were included (data from multiple time points was studied as a single entity) because the gut microbiota of human babies is very dynamic^32^,^45^. Approximately 0.5 g of each faecal sample was processed with the RNAlater protocol^46^ and stored at −70 °C prior to RNA isolation. RNA isolation was done with a protocol based on mechanical disruption^46^, after which the quality and yield of the RNA was assessed with the NanoDrop 1000 Spectrophotometer (Thermo Scientific). Sequencing was performed on a Illumina HiSeq 2500 instrument. An additional pre-sequencing rRNA removal step with the Rib-Zero kit (Epicentre) was included for sample 2D_2 . The pig metatranscriptome data were those generated from proximal colon samples of pig 2, pig 4, pig 5, pig 6, pig 8 and pig 10 from a study by Haenen *et al.*^47^, and in this study these pigs are addressed as pigs 1 to 6, respectively. It is important to note that the pigs received Neopen (MSD Animal Health) intramuscularly prior to sampling. The mice metatranscriptome data were generated from cecum samples of 15 mice from a study by Hugenholtz *et al.*^48^, and both pig and mice samples were processed and sequenced as described in this study. The sponge (*Crambe crambe*) metatranscriptome data were obtained from a sample that was collected as described before^49^. A one cm^3^ piece of sponge tissue was fixed in ten volumes of RNALater. Subsequently, RNA was extracted with a modified guanidium thiocyanate phenol-chloroform extraction method^50^ where 100 mg of tissue sample was homogenized in Solution D with glass beads in a Precellys 24 tissue homogenizer (Bertin Technologies) prior to extraction. The total RNA was treated with the Dynabeads® mRNA Purification Kit (Life Technologies) to remove sponge mRNA. One μg of the resulting RNA sample was treated with both the Epibio Ribo-Zero rRNA (Illumina) removal kit for Human/mouse/rat and the Epibio Ribo-Zero rRNA (Illumina) removal kit for meta-bacteria in order remove sponge rRNA and bacterial rRNA, respectively. Ten ng of resulting enriched bacterial mRNA was reverse-transcribed and amplified with the Ovation RNASeq system (Nugen). Sequencing was performed on a Illumina HiSeq 1000 instrument at Macrogen. In order to perform a thorough analysis for the sub-seafloor sediment, available metatranscriptome data from all six depths (≥5 metres below the seafloor) was analysed.

### Calculation of the fraction of non-ribosomal RNA reads

In order to allow for comparison of relative transcript abundance across different datasets, we determined the number of non-ribosomal RNA reads for each metatranscriptome, applying the SortMeRNA pipeline^51^, and using the included rRNA database and default filtering settings.

### Analysis of resistance gene expression

To determine the expression of antibiotic resistance genes, the metatranscriptome reads were compared with the genes present in the resistance determinants database (RED-DB, www.fibim.unisi.it/REDDB). Genes in the RED-DB are grouped according to the type of antibiotic they confer resistance to, which makes the database suitable for our purpose. Relying on the FASTA headers of resistance genes (i.e. if FASTA headers matched the string “fusion”), fusion proteins of resistance genes together with sequences unrelated to antibiotic resistance were removed from the database. Subsequently, the redundancy of the RED-DB was reduced by clustering of entries with CD-HIT-EST^52^ according to a ≥97% nucleotide identity threshold, reducing it in size to 4537 genes. Thereafter, megaBLAST^53^ was used to align the metatranscriptome reads against the non-redundant RED-DB with a nucleotide identity of ≥90% and an alignment length of ≥60 bp as alignment thresholds. For a gene to be considered expressed at least three reads were required to align with a cumulative covered gene length ≥ 200 bp. These cut-offs are more stringent than those used in previous studies^3^,^30^. When these cut-offs were made less stringent, no notable increase in the expression of antibiotic resistance genes was observed, indicating the robustness of this approach (data not shown). The hit list of resistance genes was manually inspected. Housekeeping genes that are targets of antibiotics and can become resistance genes through mutations (e.g. DNA gyrase and RNA polymerase subunit B) were removed, as well as genes that cannot always be directly associated with a resistance phenotype (e.g. *ribF*^54^ and *sanA*^55^). One-way analysis of similarities^56^ (ANOSIM, Bray-Curtis distance measure, 999 permutations) was performed with the PRIMER 6 software package (Clarke, K.R., Gorley, R.N., 2006. PRIMER v6: User Manual/Tutorial. PRIMER-E, Plymouth) to test for significant differences between the expression profiles in human adults, mice and pigs, using the cumulative relative expression levels of resistance genes against each of the 10 types of antibiotics. Rarefaction curves were prepared using the QIIME script *multiple_rarefactions.py*. The average number of resistance genes that were detected by >3 reads in the rarefied OTU tables was calculated after 1000 iterations of subsampling. Rarefaction curves were plotted and regression analysis was performed using two different equations^57^.


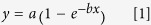



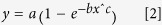


where x is the sample size (number of reads sampled), y the observed number of resistance genes and a the number of resistance genes to be expected with infinite sample size.

### Expression of secondary metabolite biosynthesis cluster genes

Various secondary metabolite biosynthesis (SMB) gene clusters encode proteins needed to produce antibiotics, and therefore their expression can give information about the potential presence of naturally produced antibiotics in a given ecological niche. For the construction of the antibiotics and secondary metabolite analysis shell (antiSMASH)^20^, a list of core domains was compiled, which are exclusively present in genes in specific types of SMB clusters. Profile Hidden Markov Models (pHMMs) are available for these domains. In order to detect the expression of SMB clusters, metatranscriptome reads were translated to protein sequences in all six open reading frames. Subsequently, the HMMer3 tool (http://hmmer.janelia.org/) was used to detect the core domains specific to the different SMB clusters. The SMB clusters for which expression was investigated included those involved in synthesis of non-ribosomal peptides, polyketides type I and II and aminoglycosides/aminocyclitols. Thresholds for domain detection were the trusted cut-offs supplied with the pHMMs, and when those were not available an e-value of 1.0 × 10^−7^ was used. Core domains were required to be detected by at least three reads in order for them to be considered expressed.

## Additional Information

**How to cite this article**: Versluis, D. *et al.* Mining microbial metatranscriptomes for expression of antibiotic resistance genes under natural conditions. *Sci. Rep.* **5**, 11981; doi: 10.1038/srep11981 (2015).

## Supplementary Material

Supplementary Information
