# Supplementary material for: Mining microbial metatranscriptomes for expression of antibiotic resistance genes under natural conditions
Source: Sci Rep. 2015 Jul 8;5:11981. doi: 10.1038/srep11981 (PMC4495384; doi:10.1038/srep11981)
Supplement: Supplementary Information [file srep11981-s1.pdf]

**Supplementary information**

# Mining microbial metatranscriptomes for expression of antibiotic resistance genes under natural conditions

**Dennis Versluis[1], Marco Maria D'Andrea[2], Javier Ramiro Garcia[1,3], Milkha M. Leimena[1,3], Floor Hugenholtz[1,3,4], Jing Zhang[1], Başak Öztürk[1], Lotta Nylund[5,7], Detmer Sipkema[1], Willem van Schaik[6], Willem M. de Vos[1,7,8], Michiel Kleerebezem[1,3,9,10], Hauke Smidt[1,3], Mark W.J. van Passel[1,11]**

[1]Laboratory of Microbiology, Wageningen University, Wageningen, The Netherlands, [2]Department of Medical Biotechnologies, University of Siena, Italy [3]Top Institute Food and Nutrition, Wageningen, The Netherlands, , [4]Netherlands Consortium for Systems Biology, University of Amsterdam, The Netherlands, [5]Functional Foods Forum, University of Turku, Turku, Finland, [6]Department of Medical Microbiology, University Medical Center Utrecht, Utrecht, The Netherlands, [7]Department of Veterinary Biosciences, University of Helsinki, Finland, [8`]Department of Bacteriology and Immunology, Haartman Institute, University of Helsinki, Finland, [9]NIZO Food Research B.V., Ede, The Netherlands, [10]Host-Microbe Interactomics Group, Wageningen University, Wageningen, The Netherlands and [11]National Institute for Public Health and the Environment, Bilthoven, The Netherlands

**A**

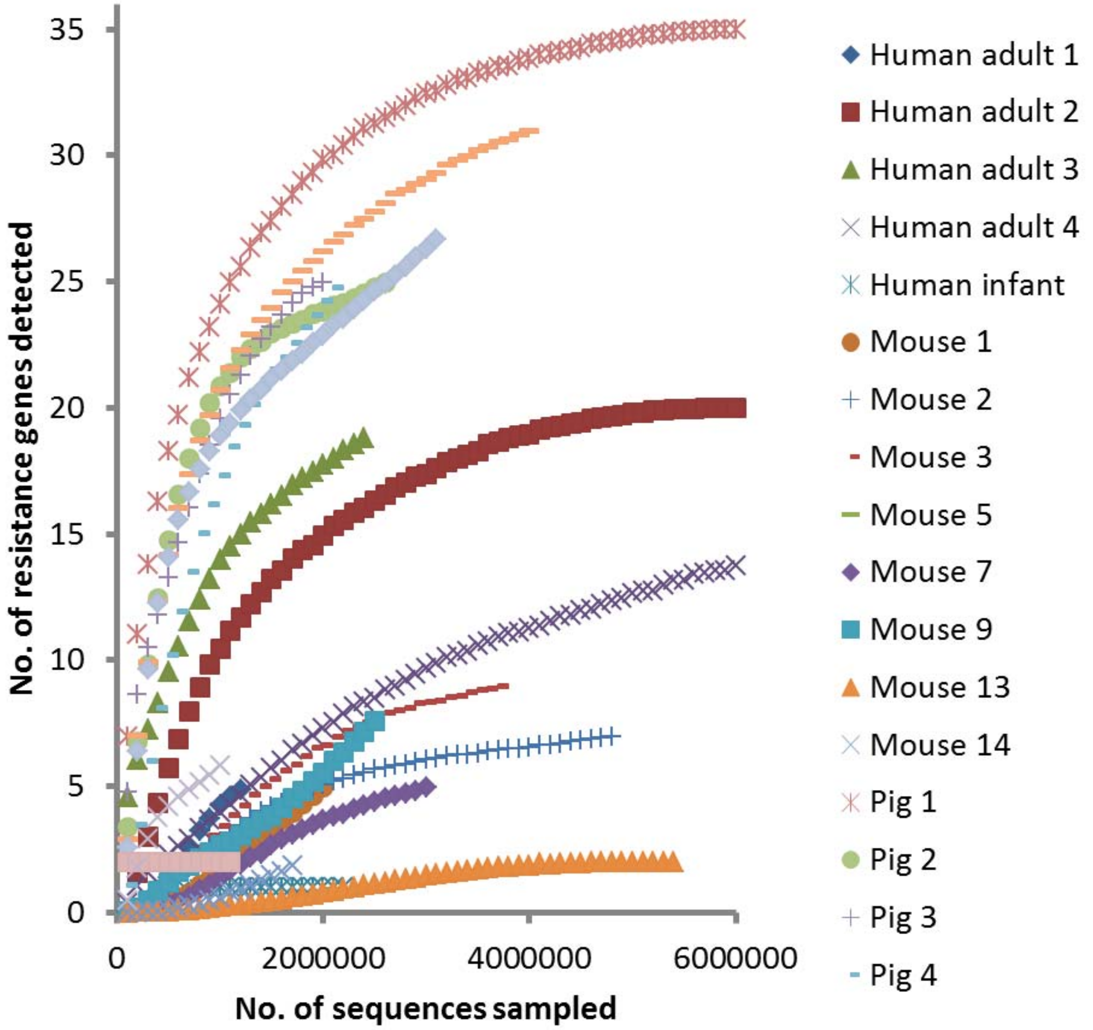

No. of resistance genes detected
35
30
25
20
15
10
5
0
0
2000000
4000000
6000000
No. of sequences sampled
Human adult 1
Human adult 2
Human adult 3
Human adult 4
Human infant
Mouse 1
Mouse 2
Mouse 3
Mouse 5
Mouse 7
Mouse 9
Mouse 13
Mouse 14
Pig 1
Pig 2
Pig 3
Pig 4

**B**

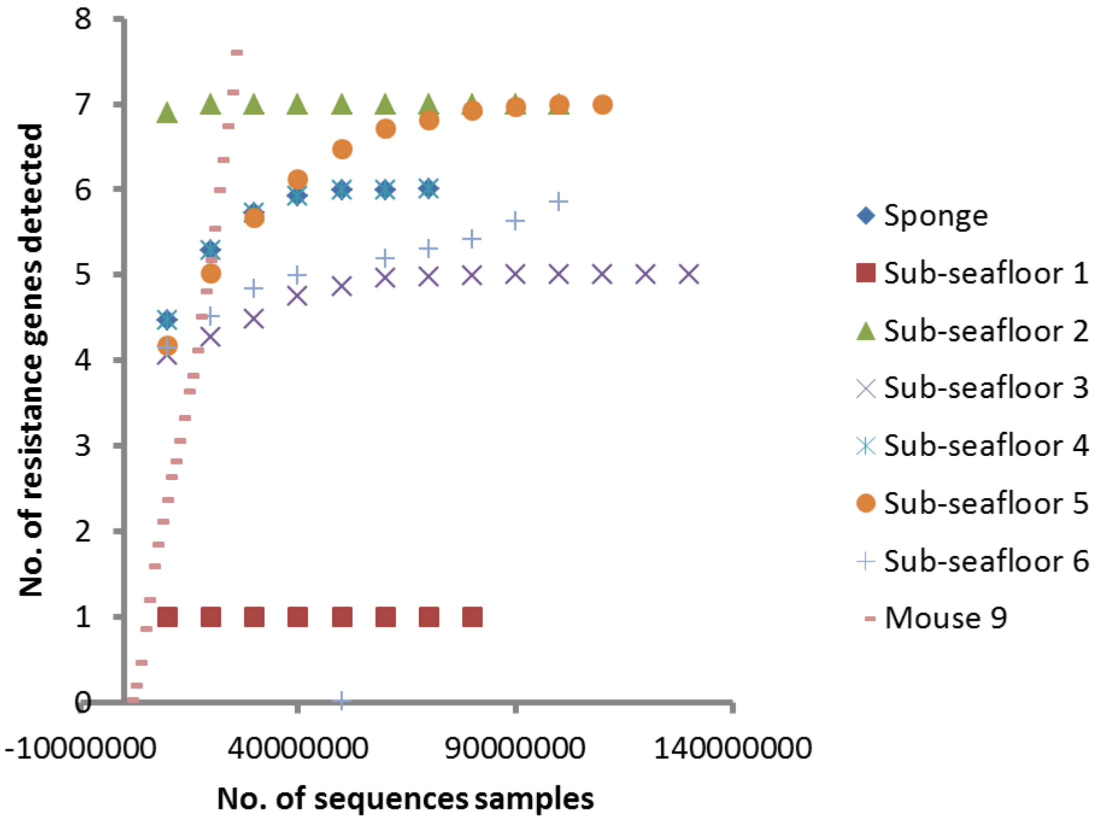


**Supplementary Figure S1** Rarefaction curves for all investigated ecological niches: (A) for datasets < 20,000,000 non-ribosomal reads (B) for datasets > 20,000,000 non-ribosomal reads.

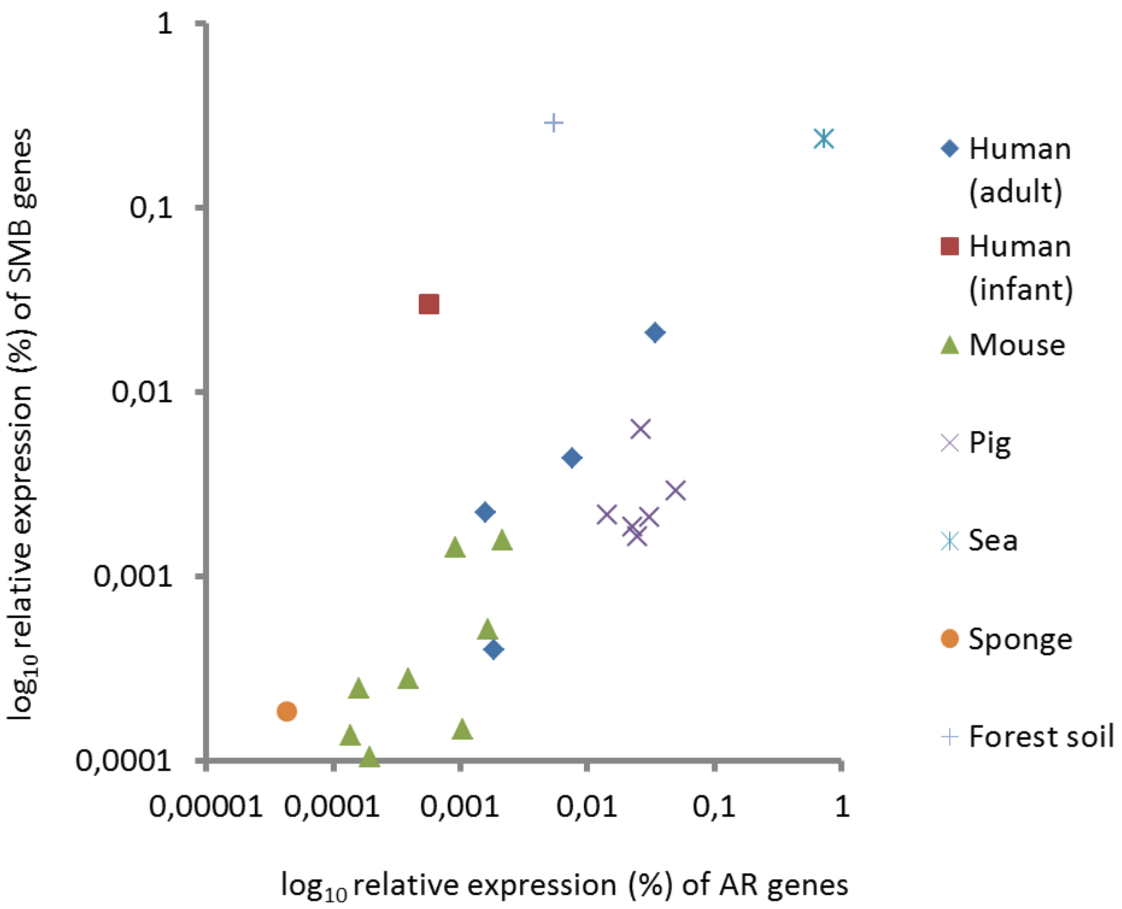


**Supplementary Figure S2** Relative cumulative expression of antibiotic resistance genes plotted against the cumulative relative expression of secondary metabolite biosynthesis domains involved in the production of type I polyketides, type II polyketides, non-ribosomal peptides and aminoglycosides.

**Supplementary Table S1** Regression analysis was done to fit the rarefaction curves using 2 equations: (f1) $y = a\,(1 - e^{-bx})$ and (f2) $y = a\,(1 - e^{-bx^c})$. Constant a indicates the number of resistance genes to be expected with infinite sample size. In the case of human 1 no good fit was obtained for equation f1.

| **Sample** | **No. of resistance genes detected** | **a (f1)** | **b (f1)** | $r^2$ | **a (f2)** | **b (f2)** | **c (f2)** | $r^2$ |
|---|---|---|---|---|---|---|---|---|
| Human 1 | 5 | n.a. | n.a. | n.a. | 5.245 | $2.05*10^{-15}$ | 2.485 | 0.9997 |
| Human 2 | 20 | 20.34 | $6.76*10^{-07}$ | 0.9962 | 20.34 | $6.49*10^{-07}$ | 1.003 | 0.9962 |
| Human 3 | 19 | 18.76 | $1.14*10^{-06}$ | 0.9064 | 24.19 | $7.25*10^{-05}$ | 0.677 | 0.9974 |
| Human 4 | 20 | 20.93 | $1.89*10^{-07}$ | 0.9956 | 20.67 | $1.49*10^{-06}$ | 0.857 | 0.9986 |
| Pig 1 | 35 | 33.86 | $1.27*10^{-06}$ | 0.9621 | 35.971 | $9.73*10^{-05}$ | 0.676 | 0.9996 |
| Pig 2 | 25 | 24.77 | $1.78*10^{-06}$ | 0.9973 | 24.456 | $6.73*10^{-07}$ | 1.075 | 0.9983 |
| Pig 3 | 25 | 26.05 | $1.46*10^{-06}$ | 0.9862 | 32.344 | $3.24*10^{-05}$ | 0.742 | 0.9972 |
| Pig 4 | 25 | 30.02 | $8.36*10^{-07}$ | 0.9957 | 26.251 | $8.34*10^{-08}$ | 1.185 | 0.9987 |
| Pig 5 | 31 | 30.10 | $1.15*10^{-06}$ | 0.9893 | 31.884 | $1.25*10^{-05}$ | 0.818 | 0.9967 |
| Pig 6 | 27 | 25.15 | $1.42*10^{-06}$ | 0.9763 | 27.235 | $2.05*10^{-05}$ | 0.791 | 0.9857 |

**Supplementary Table S2** Antibiotic resistance genes that were detected in the datasets from the different ecological niches.
Supplementary Table S4 contains the statistics and accession numbers of the datasets.

| | Resistance type | Gene description in the RED-DB | Accession | Region | No. of reads that aligned |
|---|---|---|---|---|---|
| Human adult 1 | β-lactams | blaTEM-1 | J01749 | 3293..4153 | 5 |
| | macrolides | Mel protein | NZ_ABGG01000003.1 | 85971..87434 | 6 |
| | macrolides | mefA | DQ823450.1 | 1..1218 | 5 |
| | tetracyclines | tetB | HQ652506.1 | 2100..3836 | 4 |
| | tetracyclines | multidrug ABC transporter ATPase/permease | NC_017905.1 | 1528774..1530498 | 3 |
| Human adult 2 | β-lactams | blaTEM-1 | J01749 | 3293..4153 | 57 |
| | tetracyclines | mbsA | NC_017905.1 | 1527036..1528814 | 83 |
| | tetracyclines | mdlA2 | NC_015678.1 | 963805..965529 | 48 |
| | tetracyclines | multidrug ABC transporter ATPase/permease | NC_017905.1 | 1528774..1530498 | 36 |
| | macrolides | Mel protein | NZ_ABGG01000003.1 | 85971..87434 | 30 |
| | tetracyclines | tetB | HQ652506.1 | 2100..3836 | 30 |
| | tetracyclines | tetA | HQ652506.1 | 374..2098 | 27 |
| | tetracyclines | tetM | CP002027.1 | 29270..31258 | 28 |
| | aminoglycosides | aminoglycoside 6'-N-acetyltransferase | NC_013853.1 | 1099168..1099596 | 6 |
| | macrolides | mefA | DQ823450.1 | 1..1218 | 15 |
| | tetracyclines | translation elongation factor (GTPases) | NC_009442.1 | 892240..894174 | 22 |
| | aminoglycosides | acetyltransferase, GNAT family | NZ_GG703805.1 | 94841..95269 | 4 |
| | tetracyclines | tetM | FR671403.1 | 41075..43009 | 19 |
| | tetracyclines | tetC | NC_002134.1 | 42856..43524 | 5 |
| | tetracyclines | putative translation elongation factor G | AEBQ01000040.1 | 147701..149635 | 13 |
| | tetracyclines | tetO | NC_006134.1 | 1..1920 | 13 |
| | tetracyclines | transporter, major facilitator family protein | AYGR01000226.1 | 64208..65473 | 9 |
| | tetracyclines | tet | AY196921.1 | 131..2050 | 8 |
| | tetracyclines | hypothetical protein | NZ_ABAX03000014.1 | 219600..221519 | 6 |
| | tetracyclines | tet | AY196920.1 | 1..1920 | 6 |
| Human adult 3 | β-lactams | ampC | NC_008253.1 | 4607984..4609246 | 262 |
| | macrolides | cmr | NC_004431.1 | 904537..905799 | 117 |
| | macrolides | macA | NZ_AAJU02000011.1 | 30511..31653 | 89 |
| | macrolides | ybjz | NC_007606.1 | 2184371..2186323 | 133 |
| | macrolides | macrolide transporter ATP-binding /permease protein | NC_007946.1 | 881132..883078 | 104 |
| | β-lactams | beta-lactamase | NZ_ADUB01000302.1 | 3244..4428 | 34 |
| | aminoglycosides | aminoglycoside 6'-N-acetyltransferase | NC_013853.1 | 1099168..1099596 | 8 |
| | β-lactams | blaTEM-1 | J01749 | 3293..4153 | 14 |
| | macrolides | putative membrane protein | CP000034.1 | 2186320..2187396 | 18 |
| | macrolides | macA | NZ_AAJX02000016.1 | 87441..88535 | 12 |
| | tetracyclines | tetAJ | NC_010554.1 | 2621559..2622755 | 13 |
| | aminoglycosides | acetyltransferase, GNAT family | NZ_GG703805.1 | 94841..95269 | 4 |
| | chloramphenicol | cat | M11587.1 | 880..1533 | 5 |
| | tetracyclines | msbA | NC_017905.1 | 1527036..1528814 | 11 |
| | macrolides | macrolide ABC efflux protein macB | EU199081.2 | 37973..39931 | 10 |
| | tetracyclines | multidrug ABC transporter ATPase/permease | NC_017905.1 | 1528774..1530498 | 8 |
| | macrolides | Mel protein | NZ_ABGG01000003.1 | 85971..87434 | 5 |
| | macrolides | putative drug-efflux protein | NC_012660.1 | 2819006..2820157 | 3 |
| | macrolides | mefA | DQ823450.1 | 1..1218 | 3 |
| Human adult 4 | macrolides | mefA | DQ823450.1 | 1..1218 | 34 |
| | β-lactams | blaTEM-1 | J01749 | 3293..4153 | 23 |
| | tetracyclines | msbA | NC_017905.1 | 1527036..1528814 | 48 |
| | macrolides | Mel protein | NZ_ABGG01000003.1 | 85971..87434 | 33 |
| | aminoglycosides | aminoglycoside 6'-N-acetyltransferase | NC_013853.1 | 1099168..1099596 | 8 |
| | tetracyclines | multidrug ABC transporter ATPase/permease | NC_017905.1 | 1528774..1530498 | 25 |
| | tetracyclines | mdlA2 | NC_015678.1 | 963805..965529 | 23 |
| | aminoglycosides | acetyltransferase, GNAT family | NZ_GG703805.1 | 94841..95269 | 6 |
| | tetracyclines | tetA | HQ652506.1 | 374..2098 | 18 |
| | macrolides | rRNA adenine N-6-methyltransferase | Z_JH814611.1 | 1..783 | 5 |
| | tetracyclines | tetB | HQ652506.1 | 2100..3836 | 11 |
| | β-lactams | blaTEM-116 | EU496092.1 | 80..940 | 5 |
| | tetracyclines | translation elongation factor (GTPases) | NC_009442.1 | 892240..894174 | 7 |
| | macrolides | putative drug-efflux protein | NC_012660.1 | 2819006..2820157 | 4 |
| | tetracyclines | tetM | FR671403.1 | 41075..43009 | 6 |
| | macrolides | macrolide ABC efflux protein macB | EU199081.2 | 37973..39931 | 4 |
| Human (infant) | tetracyclines | tet(32) | NZ_ACCF01000064.1 | 29230..31149 | 13 |
| Mouse 1 | tetracyclines | tetM | JGEQ01000023.1 | 4630..6555 | 7 |
| | tetracyclines | hypothetical protein | NZ_ABYH01000197.1 | 1435..3381 | 4 |
| | tetracyclines | putative translation elongation factor G | NZ_AFBR01000047.1 | 1..1878 | 4 |
| | tetracyclines | translation elongation factor G | NZ_GG698740.1 | 861124..863097 | 3 |
| | glycopeptides | vanTC | DQ022190.1 | 1802..3898 | 3 |
| Mouse 2 | tetracyclines | putative translation elongation factor G | NZ_AFBR01000047.1 | 1..1878 | 20 |
| | tetracyclines | tetM | JGEQ01000023.1 | 4630..6555 | 19 |
| | tetracyclines | translation elongation factor G | NZ_GG698740.1 | 861124..863097 | 15 |
| | tetracyclines | tetQ | AY171591.1 | 1..1313 | 8 |
| | tetracyclines | hypothetical protein | NZ_ABYH01000197.1 | 1435..3381 | 9 |
| | tetracyclines | tet(Q) | U73497.1 | 308..2320 | 6 |
| | tetracyclines | tetQ | AY171589.1 | 1..1313 | 3 |
| Mouse 3 | tetracyclines | tetM | JGEQ01000023.1 | 4630..6555 | 21 |
| | tetracyclines | translation elongation factor G | NZ_GG698740.1 | 861124..863097 | 15 |
| | β-lactams | cblA | L08472.1 | 848..1738 | 5 |
| | tetracyclines | putative translation elongation factor G | NZ_AFBR01000047.1 | 1..1878 | 9 |
| | tetracyclines | tetW | NC_008011.1 | 225939..227858 | 9 |
| | tetracyclines | hypothetical protein | NZ_ABYH01000197.1 | 1435..3381 | 8 |
| | tetracyclines | tetQ | AY171589.1 | 1..1313 | 5 |
| | tetracyclines | elongation factor Tu GTP binding domain-containing protein | NC_016610.1 | 1331023-1332960 | 6 |
| | tetracyclines | MDR-type permease, probably tetracycline resistance protein | NZ_ABYJ02000150.1 | 1437..2675 | 3 |
| Mouse 4 | - | | | | |
| Mouse 5 | tetracyclines | MDR-type permease, probably tetracycline resistance protein | NZ_ABYJ02000150.1 | 1437..2675 | 3 |
| Mouse 6 | - | | | | |
| Mouse 7 | tetracyclines | tetM | JGEQ01000023.1 | 4630..6555 | 8 |
| | tetracyclines | translation elongation factor G | NZ_GG698740.1 | 861124..863097 | 7 |
| | tetracyclines | putative translation elongation factor G | NZ_AFBR01000047.1 | 1..1878 | 6 |

| | | | | | |
|---|---|---|---|---|---|
| | tetracyclines | tetQ | AY171591.1 | 1..1313 | 3 |
| | tetracyclines | tetW | NC_008011.1 | 225939..227858 | 4 |
| Mouse 8 | - | | | | |
| Mouse 9 | tetracyclines | tetM | JGEQ01000023.1 | 4630..6555 | 15 |
| | tetracyclines | putative translation elongation factor G | NZ_AFBR01000047.1 | 1..1878 | 14 |
| | aminoglycosides | NptII | GQ463722.1 | 5456-6463 | 4 |
| | tetracyclines | translation elongation factor G | NZ_GG698740.1 | 861124..863097 | 5 |
| | macrolides | hypothetical protein | ACTV01000015.1 | 147457..148674 | 3 |
| | tetracyclines | flavoprotein | NZ_EQ973254.1 | 112027..113223 | 3 |
| | tetracyclines | hypothetical protein | NZ_AAYH02000047.1 | 190757..192022 | 3 |
| | tetracyclines | teto | NC_006134.1 | 1..1920 | 3 |
| Mouse 10 | - | | | | |
| Mouse 11 | - | | | | |
| Mouse 12 | - | | | | |
| Mouse 13 | tetracyclines | putative translation elongation factor G | NZ_AFBR01000047.1 | 1..1878 | 6 |
| | tetracyclines | tetM | JGEQ01000023.1 | 4630..6555 | 5 |
| Mouse 14 | tetracyclines | putative translation elongation factor G | NZ_AFBR01000047.1 | 1..1878 | 4 |
| | tetracyclines | tetM | JGEQ01000023.1 | 4630..6555 | 3 |
| Mouse 15 | - | | | | |
| Pig 1 | tetracyclines | tetM | JGEQ01000023.1 | 4630..6555 | 512 |
| | tetracyclines | tetQ | AY171589.1 | 1..1313 | 293 |
| | tetracyclines | translation elongation factor G | NZ_GG698740.1 | 861124..863097 | 375 |
| | tetracyclines | small GTP-binding protein domain | FP929044.1 | 831854..833806 | 271 |
| | tetracyclines | putative translation elongation factor G | NZ_AFBR01000047.1 | 1..1878 | 249 |
| | tetracyclines | hypothetical protein | NZ_ABYH01000197.1 | 1435..3381 | 218 |
| | tetracyclines | tet(Q) | U73497.1 | 308..2320 | 171 |
| | tetracyclines | tet(W) | EF177463.1 | 6391..8313 | 148 |
| | tetracyclines | tet(W/32/O) | AM710602.1 | 1..1920 | 116 |
| | aminoglycosides | Aad9 | AY701528.1 | 6015..6791 | 45 |
| | macrolides | H+ Antiporter protein | NZ_ACTC01000108.1 | 28520..29746 | 68 |
| | macrolides | transporter, permease component | NC_009706.1 | 2693792..2695018 | 65 |
| | tetracyclines | tetQ | AY171592.1 | 1..1313 | 69 |
| | tetracyclines | MDR-type permease, probably tetracycline resistance protein | NZ_ABYJ02000150.1 | 1437..2675 | 58 |
| | macrolides | hypothetical protein | ACTV01000015.1 | 147457..148674 | 56 |
| | β-lactams | cfxA4 | AY769933.1 | 1..966 | 38 |
| | β-lactams | blaACI-1 | AJ007350.1 | 240..1094 | 29 |
| | tetracyclines | tetQ | AY171591.1 | 1..1313 | 36 |
| | tetracyclines | tetW | NC_008011.1 | 225939..227858 | 38 |
| | aminoglycosides | aphA-3 | AJ489618.1 | 1356..2141 | 15 |
| | macrolides | rRNA adenine N-6-methyltransferase | Z_JH814611.1 | 1..783 | 13 |
| | macrolides | mef | EU870856.1 | 1..1218 | 19 |
| | tetracyclines | tet(44) | NZ_ABDU01000081.2 | 172599..174521 | 29 |
| | macrolides | mef(A) | NC_006086.1 | 1167541..1168758 | 17 |
| | chloramphenicol | cat | AB289766.1 | 5228..5908 | 9 |
| | tetracyclines | elongation factor Tu GTP binding domain-containing protein | NC_016610.1 | 1331023..1332960 | 27 |
| | aminoglycosides | ant(6)-Ia | AY701528.1 | 492..1358 | 9 |
| | tetracyclines | tet | AY196920.1 | 1..1920 | 16 |
| | aminoglycosides | aadE | FN568063.1 | 1762050..1762958 | 7 |
| | tetracyclines | teto | NC_006134.1 | 1..1920 | 11 |
| | tetracyclines | tet | AY196921.1 | 131..2050 | 11 |
| | tetracyclines | hypothetical protein | NZ_ABAX03000014.1 | 219600..221519 | 8 |
| | tetracyclines | tet(X) | NZ_EQ973223.1 | 10935-12101 | 4 |
| | tetracyclines | tetOW | AY485122.1 | 1..1920 | 5 |
| | tetracyclines | small GTP-binding protein domain | FP929050.1 | 2873814-2875733 | 4 |
| Pig 2 | tetracyclines | MDR-type permease, probably tetracycline resistance protein | NZ_ABYJ02000150.1 | 1437..2675 | 110 |
| | tetracyclines | tetM | JGEQ01000023.1 | 4630..6555 | 124 |
| | tetracyclines | small GTP-binding protein domain | FP929044.1 | 831854..833806 | 74 |
| | tetracyclines | putative translation elongation factor G | NZ_AFBR01000047.1 | 1..1878 | 60 |
| | aminoglycosides | Aad9 | AY701528.1 | 6015..6791 | 23 |
| | tetracyclines | tetQ | AY171591.1 | 1..1313 | 32 |
| | tetracyclines | hypothetical protein | NZ_ABYH01000197.1 | 1435..3381 | 36 |
| | tetracyclines | tet(Q) | U73497.1 | 308-2320 | 34 |
| | aminoglycosides | ant(6)-Ia | AY701528.1 | 492..1358 | 14 |
| | macrolides | hypothetical protein | ACTV01000015.1 | 147457..148674 | 20 |
| | macrolides | transporter, permease component | NC_009706.1 | 2693792..2695018 | 18 |
| | macrolides | H+ Antiporter protein | NZ_ACTC01000108.1 | 28520..29746 | 16 |
| | tetracyclines | tetQ | AY171589.1 | 1..1313 | 15 |
| | tetracyclines | translation elongation factor G | NZ_GG698740.1 | 861124-863097 | 21 |
| | tetracyclines | tetQ | AY171592.1 | 1..1313 | 12 |
| | β-lactams | cfxA4 | AY769933.1 | 1..966 | 9 |
| | tetracyclines | tet(W) | EF177463.1 | 6391..8313 | 16 |
| | tetracyclines | tet | AY196920.1 | 1..1920 | 15 |
| | macrolides | rRNA adenine N-6-methyltransferase | Z_JH814611.1 | 1..783 | 6 |
| | tetracyclines | tet(W/32/O) | AM710602.1 | 1..1920 | 12 |
| | macrolides | mef | EU870856.1 | 1..1218 | 7 |
| | tetracyclines | hypothetical protein | NZ_ABAX03000014.1 | 219600..221519 | 10 |
| | tetracyclines | teto | NC_006134.1 | 1..1920 | 9 |
| | aminoglycosides | aadE | FN568063.1 | 1762050..1762958 | 3 |
| | tetracyclines | elongation factor Tu GTP binding domain-containing protein | NC_016610.1 | 1331023..1332960 | 3 |
| Pig 3 | tetracyclines | tetM | JGEQ01000023.1 | 4630..6555 | 106 |
| | tetracyclines | tetQ | AY171589.1 | 1..1313 | 65 |
| | tetracyclines | MDR-type permease, probably tetracycline resistance protein | NZ_ABYJ02000150.1 | 1437..2675 | 45 |
| | tetracyclines | putative translation elongation factor G | NZ_AFBR01000047.1 | 1..1878 | 56 |
| | tetracyclines | small GTP-binding protein domain | FP929044.1 | 831854..833806 | 58 |
| | tetracyclines | translation elongation factor G | NZ_GG698740.1 | 861124..863097 | 54 |
| | tetracyclines | hypothetical protein | NZ_ABYH01000197.1 | 1435..3381 | 48 |
| | tetracyclines | tet(W) | EF177463.1 | 6391..8313 | 42 |
| | tetracyclines | tet(Q) | U73497.1 | 308..2320 | 38 |
| | tetracyclines | tetQ | AY171592.1 | 1..1313 | 16 |
| | β-lactams | cfxA4 | AY769933.1 | 1..966 | 11 |
| | β-lactams | blaACI-1 | AJ007350.1 | 240..1094 | 7 |
| | macrolides | transporter, permease component | NC_009706.1 | 2693792..2695018 | 9 |

| | | | | | |
|---|---|---|---|---|---|
| | macrolides | H+ Antiporter protein | NZ_ACTC01000108.1 | 28520..29746 | 9 |
| | aminoglycosides | Aad9 | AY701528.1 | 6015..6791 | 5 |
| | tetracyclines | tetQ | AY171591.1 | 1..1313 | 9 |
| | macrolides | rRNA adenine N-6-methyltransferase | Z_JH814611.1 | 1..783 | 4 |
| | tetracyclines | teto | NC_006134.1 | 1..1920 | 10 |
| | macrolides | hypothetical protein | ACTV01000015.1 | 147457-148674 | 5 |
| | tetracyclines | tet(W/32/O) | AM710602.1 | 1..1920 | 7 |
| | tetracyclines | tet | AY196920.1 | 1..1920 | 7 |
| | tetracyclines | elongation factor Tu GTP binding domain-containing protein | NC_016610.1 | 1331023..1332960 | 6 |
| | tetracyclines | tet | AY196921.1 | 131..2050 | 4 |
| | tetracyclines | hypothetical protein | NZ_ABAX03000014.1 | 219600..221519 | 4 |
| | tetracyclines | tet(W) | AY049983.2 | 8309..10228 | 3 |
| Pig 4 | tetracyclines | MDR-type permease, probably tetracycline resistance protein | NZ_ABYJ02000150.1 | 1437..2675 | 45 |
| | tetracyclines | small GTP-binding protein domain | FP929044.1 | 831854..833806 | 42 |
| | aminoglycosides | Aad9 | AY701528.1 | 6015..6791 | 10 |
| | tetracyclines | tet | AY196920.1 | 1..1920 | 25 |
| | aminoglycosides | ant(6)-Ia | AY701528.1 | 492..1358 | 10 |
| | tetracyclines | putative translation elongation factor G | NZ_AFBR01000047.1 | 1..1878 | 21 |
| | tetracyclines | tetM | JGEQ01000023.1 | 4630..6555 | 21 |
| | tetracyclines | teto | NC_006134.1 | 1..1920 | 17 |
| | tetracyclines | tet(W) | EF177463.1 | 6391..8313 | 17 |
| | β-lactams | cfxA4 | AY769933.1 | 1..966 | 7 |
| | tetracyclines | tet(W/32/O) | AM710602.1 | 1..1920 | 12 |
| | macrolides | hypothetical protein | ACTV01000015.1 | 147457..148674 | 8 |
| | tetracyclines | tet | AY196921.1 | 131..2050 | 12 |
| | aminoglycosides | streptothricin acetyltransferase | NC_005000.1 | 8232..8762 | 3 |
| | aminoglycosides | aphA-3 | AJ489618.1 | 1356..2141 | 5 |
| | tetracyclines | tetA(P) | NC_010937.1 | 559..1821 | 7 |
| | macrolides | transporter, permease component | NC_009706.1 | 2693792..2695018 | 7 |
| | tetracyclines | hypothetical protein | NZ_ABAX03000014.1 | 219600..221519 | 10 |
| | tetracyclines | translation elongation factor G | NZ_GG698740.1 | 861124..863097 | 6 |
| | tetracyclines | tet(Q) | U73497.1 | 308..2320 | 6 |
| | tetracyclines | small GTP-binding protein domain | FP929050.1 | 2873814..2875733 | 5 |
| | tetracyclines | tetQ | AY171592.1 | 1..1313 | 3 |
| | tetracyclines | hypothetical protein | NZ_ABYH01000197.1 | 1435-3381 | 4 |
| | tetracyclines | tet(32) | NZ_ACCF01000064.1 | 29230..31149 | 3 |
| | tetracyclines | tet(44) | NZ_ABDU01000081.2 | 172599..174521 | 3 |
| Pig 5 | tetracyclines | MDR-type permease, probably tetracycline resistance protein | NZ_ABYJ02000150.1 | 1437..2675 | 131 |
| | aminoglycosides | Aad9 | AY701528.1 | 6015..6791 | 64 |
| | tetracyclines | tetM | JGEQ01000023.1 | 4630..6555 | 121 |
| | tetracyclines | small GTP-binding protein domain | FP929044.1 | 831854..833806 | 97 |
| | tetracyclines | putative translation elongation factor G | NZ_AFBR01000047.1 | 1..1878 | 73 |
| | tetracyclines | tet(W) | EF177463.1 | 6391..8313 | 69 |
| | tetracyclines | hypothetical protein | NZ_ABYH01000197.1 | 1435..3381 | 61 |
| | aminoglycosides | ant(6)-Ia | AY701528.1 | 492..1358 | 26 |
| | aminoglycosides | aphA-3 | AJ489618.1 | 1356..2141 | 24 |
| | tetracyclines | tetQ | AY171591.1 | 1..1313 | 30 |
| | aminoglycosides | aadE | FN568063.1 | 1762050..1762958 | 20 |
| | β-lactams | cfxA4 | AY769933.1 | 1..966 | 21 |
| | tetracyclines | elongation factor Tu GTP binding domain-containing protein | NC_016610.1 | 1331023..1332960 | 39 |
| | tetracyclines | tet(Q) | U73497.1 | 308..2320 | 42 |
| | β-lactams | blaACI-1 | AJ007350.1 | 240..1094 | 14 |
| | tetracyclines | tet(W/32/O) | AM710602.1 | 1..1920 | 22 |
| | tetracyclines | tetQ | AY171592.1 | 1..1313 | 14 |
| | macrolides | hypothetical protein | ACTV01000015.1 | 147457..148674 | 13 |
| | tetracyclines | translation elongation factor G | NZ_GG698740.1 | 861124..863097 | 20 |
| | tetracyclines | tet | AY196920.1 | 1..1920 | 17 |
| | macrolides | transporter, permease component | NC_009706.1 | 2693792..2695018 | 8 |
| | tetracyclines | tetQ | AY171589.1 | 1..1313 | 9 |
| | tetracyclines | teto | NC_006134.1 | 1..1920 | 11 |
| | macrolides | ribosomal RNA adenine dimethylase family protein | AFBR01000078.1 | 2404..3263 | 5 |
| | tetracyclines | tet | AY196921.1 | 131..2050 | 9 |
| | tetracyclines | mef(A) | NC_006086.1 | 1167541..168758 | 5 |
| | macrolides | H+ Antiporter protein | NZ_ACTC01000108.1 | 28520..29746 | 5 |
| | tetracyclines | tetW | NC_008011.1 | 225939..227858 | 5 |
| | β-lactams | Beta-lactamase class D | FP929042.1 | 1086188..1087984 | 4 |
| | tetracyclines | tetOW | AY485122.1 | 1..1920 | 3 |
| | tetracyclines | hypothetical protein | NZ_ABAX03000014.1 | 219600..221519 | 3 |
| Pig 6 | tetracyclines | tetM | JGEQ01000023.1 | 4630..6555 | 139 |
| | tetracyclines | MDR-type permease, probably tetracycline resistance protein | NZ_ABYJ02000150.1 | 1437..2675 | 61 |
| | tetracyclines | putative translation elongation factor G | NZ_AFBR01000047.1 | 1..1878 | 71 |
| | tetracyclines | translation elongation factor G | NZ_GG698740.1 | 861124..863097 | 62 |
| | tetracyclines | tetQ | AY171589.1 | 1..1313 | 35 |
| | tetracyclines | hypothetical protein | NZ_ABYH01000197.1 | 1435..3381 | 43 |
| | tetracyclines | tetQ | AY171591.1 | 1..1313 | 29 |
| | tetracyclines | small GTP-binding protein domain | FP929044.1 | 831854..833806 | 38 |
| | tetracyclines | tetQ | AY171592.1 | 1..1313 | 24 |
| | tetracyclines | tet(W) | EF177463.1 | 6391..8313 | 33 |
| | macrolides | H+ Antiporter protein | NZ_ACTC01000108.1 | 28520..29746 | 20 |
| | β-lactams | cfxA4 | AY769933.1 | 1..966 | 17 |
| | tetracyclines | tet(Q) | U73497.1 | 308..2320 | 30 |
| | macrolides | hypothetical protein | ACTV01000015.1 | 147457..148674 | 18 |
| | tetracyclines | tet(W/32/O) | AM710602.1 | 1..1920 | 24 |
| | macrolides | transporter, permease component | NC_009706.1 | 2693792..2695018 | 11 |
| | tetracyclines | tet | AY196920.1 | 1..1920 | 18 |
| | aminoglycosides | ant(6)-Ia | AY701528.1 | 492..1358 | 5 |
| | tetracyclines | teto | NC_006134.1 | 1..1920 | 11 |
| | tetracyclines | tet | AY196921.1 | 131..2050 | 10 |
| | macrolides | ribosomal RNA adenine dimethylase family protein | AFBR01000078.1 | 2404..3263 | 4 |
| | tetracyclines | small GTP-binding protein domain | FP929050.1 | 2873814..2875733 | 7 |
| | aminoglycosides | streptomycin 3''-adenylyltransferase | NZ_GG688629.1 | 556503..557312 | 3 |
| | tetracyclines | hypothetical protein | NZ_ABAX03000014.1 | 219600..221519 | 5 |

| | | | | | |
|---|---|---|---|---|---|
| | macrolides | mef | EU870856.1 | 1..1218 | 3 |
| | tetracyclines | tet(W) | AY049983.2 | 8309..10228 | 3 |
| | tetracyclines | elongation factor Tu GTP binding domain-containing protein | NC_016610.1 | 1331023..1332960 | 3 |
| Sea bacterioplankton | β-lactams | blaTEM-1 | J01749 | 3293..4153 | 7725 |
| | β-lactams | blaTEM-116 | EU496092.1 | 80..940 | 537 |
| Sponge | β-lactams | blaTEM-1 | J01749 | 3293..4153 | 65 |
| | β-lactams | blaTEM-116 | EU496092.1 | 80..940 | 15 |
| Forest soil | chloramphenicol | cat | AF187996.1 | 4225..4986 | 12 |
| | tetracyclines | tetracycline resistance structural protein TetA | NZ_ACUI01000001.1 | 170717..171907 | 19 |
| | β-lactams | blaTEM-1 | J01749 | 3293..4153 | 11 |
| | tetracyclines | tetracycline resistance protein | NZ_ACBP01000105.1 | 106..1254 | 10 |
| | aminoglycosides | kanR | DQ333233.1 | 2052..2870 | 4 |
| | chloramphenicol | hypothetical protein | NZ_AAVO02000057.1 | 111.850 | 3 |
| Sub-seafloor (5 mbsf) | | blaTEM-1 | J01749 | 3293..4153 | 86 |
| Sub-seafloor (30 mbsf) | β-lactams | blaTEM-1 | J01749 | 3293..4153 | 4486 |
| | aminoglycosides | aph | 2:888_FJ155667.1 | 1519..2373 | 1495 |
| | tetracyclines | tetracycline resistance protein | NZ_ACBP01000105.1 | 106..1254 | 742 |
| | aminoglycosides | neo | NC_022344.1 | 246279..247073 | 491 |
| | β-lactams | blaTEM-116 | EU496092.1 | 80..940 | 496 |
| | tetracyclines | tet(39) | AY743590.1 | 749..1936 | 488 |
| | tetracyclines | tet(C) | EU496095.1 | 90..1280 | 54 |
| Sub-seafloor (50 mbsf) | chloramphenicol | cat | AB289766.1 | 5228..5908 | 9648 |
| | β-lactams | blaTEM-1 | J01749 | 3293..4153 | 535 |
| | chloramphenicol | hypothetical protein | NZ_AAVO02000057.1 | 111.850 | 296 |
| | chloramphenicol | cat | AF187996.1 | 4225..4986 | 107 |
| | chloramphenicol | CmR | GU574771.1 | 4400..5110 | 17 |
| | aminoglycosides | aph | FJ155667.1 | 1519..2373 | 9 |
| Sub-seafloor (70 mbsf) | aminoglycosides | aac(6')-Ix | AF031332.1 | 1..441 | 8654 |
| | aminoglycosides | aac(6')-Iu | AF031329.1 | 1..441 | 1413 |
| | aminoglycosides | aac(6')-Is | AF031327.1 | 1..441 | 530 |
| | β-lactams | blaOXA-212 | JN861780.1 | 1..825 | 202 |
| | β-lactams | beta-lactamase | NC_010678.1 | 66041..66868 | 12 |
| Sub-seafloor (91 mbsf) | aminoglycosides | aac(6')-Ix | AF031332.1 | 1..441 | 5257 |
| | aminoglycosides | aac(6')-Iu | AF031329.1 | 1..441 | 560 |
| | β-lactams | Beta-lactamase | NC_013446.1 | 2966741..2967490 | 44 |
| | β-lactams | beta-lactamase | NC_010678.1 | 66041..66868 | 22 |
| | β-lactams | blaOXA-60d | AY664506.1 | 1..816 | 9 |
| | β-lactams | blaTEM-1 | J01749 | 3293..4153 | 6 |
| | macrolides | multidrug efflux system transmembrane protein | NC_009659.1 | 1198998..1200710 | 13 |
| Sub-seafloor (159 mbsf) | aminoglycosides | aac(6')-Ix | AF031332.1 | 1..441 | 3121 |
| | aminoglycosides | aac(6')-Iu | AF031329.1 | 1..441 | 135 |
| | aminoglycosides | aadA7 | DQ520937.1 | 593..1390 | 172 |
| | aminoglycosides | strA | CT025832.1 | 22279..23115 | 94 |
| | β-lactams | beta-lactamase | NC_010678.1 | 66041..66868 | 14 |
| | β-lactams | bla | NC_004463.1 | 1030253..1029225 | 3 |

**Supplementary Table S3** Number of reads with which domains were detected that can be relevant to antibiotic biosynthesis (for detailed information about the datasets, see Supplementary Table S4).

| | | | *Human (adult)* | | | | *Human (infant)* | | | | *Mouse* | | | | | | | | | | | | | | | *Pig* | | | | | | *Sea* | *Sponge* | *Forest soil* | *Sub-seafloor* | | | | | |
|---|---|---|---|---|---|---|---|---|---|---|---|---|---|---|---|---|---|---|---|---|---|---|---|---|---|---|---|---|---|---|---|---|---|---|---|---|---|---|---|---|
| | | *Domain info* | *1* | *2* | *3* | *4* | *1 (t=1)* | *1 (t=2 (1))* | *1 (t=2 (2))* | *1 (t=3)* | *1* | *2* | *3* | *4* | *5* | *6* | *7* | *8* | *9* | *10* | *11* | *12* | *13* | *14* | *15* | *1* | *2* | *3* | *4* | *5* | *6* | *Bacteriopl.* | | | *5 mbsf* | *30 mbsf* | *50 mbsf* | *70 mbsf* | *91 mbsf* | *159 mbsf* |
| *Type I polyketide* | mod_KS | Modular ketosynthase | 0 | 0 | 85 | 0 | 0 | 0 | 0 | 0 | 0 | 0 | 0 | 0 | 0 | 0 | 0 | 0 | 0 | 0 | 0 | 0 | 0 | 0 | 0 | 0 | 7 | 0 | 0 | 0 | 0 | 106 | 0 | 150 | 0 | 565 | 18 | 297 | 41 | 359 |
| *synthesis* | hyb_KS | Hybrid ketosynthase | 0 | 0 | 102 | 0 | 0 | 0 | 0 | 0 | 0 | 0 | 0 | 0 | 0 | 0 | 0 | 0 | 0 | 0 | 0 | 0 | 0 | 0 | 0 | 0 | 16 | 0 | 0 | 0 | 0 | 123 | 0 | 149 | 0 | 947 | 14 | 95 | 39 | 479 |
| | ene_KS | Enediyine ketosynthase | 0 | 0 | 0 | 0 | 0 | 0 | 0 | 0 | 0 | 0 | 0 | 0 | 0 | 0 | 0 | 0 | 0 | 0 | 0 | 0 | 0 | 0 | 0 | 0 | 0 | 0 | 0 | 0 | 0 | 37 | 0 | 102 | 0 | 0 | 0 | 0 | 0 | 0 |
| | tra_KS | Trans-AT ketosynthase | 0 | 0 | 94 | 0 | 0 | 0 | 0 | 0 | 0 | 0 | 0 | 0 | 0 | 0 | 0 | 0 | 0 | 0 | 0 | 0 | 0 | 0 | 0 | 0 | 17 | 0 | 0 | 0 | 0 | 105 | 0 | 159 | 0 | 373 | 0 | 6 | 32 | 456 |
| | itr_KS | Iterative ketosynthase | 0 | 0 | 0 | 0 | 0 | 0 | 4 | 0 | 0 | 0 | 0 | 0 | 0 | 0 | 0 | 0 | 0 | 0 | 0 | 0 | 0 | 0 | 0 | 0 | 12 | 0 | 0 | 0 | 0 | 165 | 0 | 172 | 0 | 124 | 0 | 5 | 38 | 560 |
| | PKS_KS | Ketosynthase domain | 0 | 0 | 105 | 0 | 0 | 0 | 0 | 0 | 0 | 0 | 0 | 0 | 0 | 0 | 0 | 0 | 0 | 0 | 0 | 0 | 0 | 0 | 0 | 0 | 23 | 0 | 0 | 0 | 0 | 160 | 3 | 174 | 0 | 824 | 14 | 20 | 57 | 668 |
| | PKS_AT | Acyltransferase domain | 0 | 0 | 22 | 0 | 0 | 0 | 5 | 0 | 0 | 0 | 0 | 0 | 0 | 0 | 0 | 0 | 0 | 0 | 0 | 0 | 0 | 0 | 0 | 0 | 0 | 0 | 0 | 0 | 0 | 87 | 0 | 95 | 0 | 0 | 0 | 0 | 0 | 46 |
| *Type II polyketide* | t2clf | Type II PKS Chain length factor | 0 | 0 | 0 | 0 | 0 | 0 | 13 | 0 | 0 | 0 | 0 | 0 | 0 | 0 | 0 | 0 | 0 | 0 | 0 | 0 | 0 | 0 | 0 | 0 | 0 | 0 | 0 | 0 | 0 | 194 | 0 | 156 | 0 | 0 | 0 | 0 | 0 | 0 |
| *synthesis* | t2ks | Type II PKS ketosynthase | 0 | 0 | 0 | 0 | 0 | 0 | 58 | 0 | 0 | 0 | 0 | 4 | 0 | 3 | 7 | 0 | 0 | 0 | 0 | 0 | 0 | 0 | 0 | 7 | 12 | 3 | 4 | 0 | 4 | 416 | 0 | 216 | 16 | 9 | 9 | 0 | 14 | 13 |
| | t2pks2 | Type II PKS ketosynthase, model 2 | 0 | 17 | 0 | 4 | 0 | 0 | 56 | 0 | 3 | 8 | 28 | 4 | 3 | 10 | 23 | 4 | 13 | 0 | 3 | 0 | 6 | 5 | 0 | 39 | 30 | 13 | 11 | 8 | 11 | 392 | 0 | 203 | 311 | 181 | 133 | 8 | 95 | 97 |
| *Non-ribosomal peptide* | A-OX | Adenylation domain with integrated oxidase | 0 | 0 | 6 | 0 | 0 | 0 | 31 | 0 | 0 | 0 | 0 | 0 | 0 | 0 | 0 | 0 | 0 | 0 | 0 | 0 | 0 | 0 | 0 | 0 | 4 | 0 | 0 | 0 | 0 | 20 | 0 | 318 | 10 | 506 | 1961 | 6 | 10 | 45 |
| *synthesis* | AMP-binding | Adenylation domain | 5 | 55 | 57 | 157 | 0 | 0 | 123 | 0 | 0 | 3 | 8 | 0 | 0 | 4 | 8 | 0 | 8 | 0 | 0 | 0 | 0 | 0 | 0 | 25 | 11 | 10 | 11 | 11 | 8 | 615 | 269 | 977 | 221 | 759 | 3961 | 312 | 919 | 799 |
| | Condensation | Condensation domain | 0 | 0 | 0 | 0 | 0 | 0 | 0 | 0 | 0 | 0 | 0 | 0 | 0 | 0 | 0 | 0 | 0 | 0 | 0 | 0 | 0 | 0 | 0 | 0 | 0 | 0 | 0 | 0 | 0 | 5 | 0 | 81 | 0 | 0 | 0 | 0 | 0 | 0 |
| *Aminoglycoside/* | ValA | ValA-like 2-epi-5-epi-valiolone synthase | 0 | 0 | 0 | 0 | 0 | 0 | 0 | 0 | 0 | 0 | 0 | 0 | 0 | 0 | 0 | 0 | 0 | 0 | 0 | 0 | 0 | 0 | 0 | 0 | 0 | 0 | 0 | 0 | 0 | 37 | 0 | 19 | 0 | 0 | 0 | 0 | 13 | 16 |
| *aminocyclitol* | salQ_like | 2-epi-5-epi-valiolone synthase, SalQ-like | 0 | 0 | 0 | 0 | 0 | 0 | 4 | 0 | 0 | 0 | 0 | 0 | 0 | 0 | 0 | 0 | 0 | 0 | 0 | 0 | 0 | 0 | 0 | 0 | 0 | 0 | 0 | 0 | 0 | 59 | 0 | 24 | 0 | 0 | 0 | 0 | 36 | 86 |
| *synthesis* | SpcDK_glyc_like | SpcD-/SpcK-like thymidylyltransferas | 0 | 193 | 34 | 234 | 0 | 0 | 140 | 0 | 0 | 14 | 23 | 0 | 0 | 8 | 6 | 3 | 43 | 0 | 6 | 4 | 0 | 0 | 0 | 105 | 32 | 16 | 20 | 47 | 36 | 65 | 0 | 39 | 24 | 327 | 3326 | 25 | 254 | 440 |
| | DOIS | 2-deoxy-scyllo-inosose synthase | 0 | 0 | 0 | 0 | 0 | 0 | 5 | 0 | 0 | 0 | 0 | 0 | 0 | 0 | 0 | 0 | 0 | 0 | 0 | 0 | 0 | 0 | 0 | 0 | 0 | 0 | 0 | 0 | 0 | 69 | 0 | 39 | 42 | 5 | 7 | 0 | 26 | 62 |
| | NeoL_like | NeoL-like deacetylase | 0 | 0 | 0 | 0 | 0 | 0 | 0 | 0 | 0 | 0 | 0 | 0 | 0 | 0 | 0 | 0 | 0 | 0 | 0 | 0 | 0 | 0 | 0 | 0 | 0 | 0 | 0 | 0 | 0 | 0 | 0 | 0 | 0 | 0 | 0 | 0 | 0 | 0 |
| | strK1_like | StrK-like phosphatase | 0 | 0 | 8 | 0 | 0 | 0 | 0 | 0 | 0 | 0 | 0 | 0 | 0 | 0 | 0 | 0 | 0 | 0 | 0 | 0 | 0 | 0 | 0 | 0 | 0 | 0 | 0 | 0 | 0 | 7 | 0 | 0 | 0 | 0 | 0 | 7 | 27 | 15 |
| | strH_like | StrH-like glycosyltransferase | 0 | 0 | 0 | 0 | 0 | 0 | 0 | 0 | 0 | 0 | 0 | 0 | 0 | 0 | 0 | 0 | 0 | 0 | 0 | 0 | 0 | 0 | 0 | 0 | 0 | 0 | 0 | 0 | 0 | 0 | 0 | 0 | 0 | 0 | 0 | 0 | 0 | 0 |
| | strK2_like | StrK-like phosphatase | 0 | 0 | 0 | 0 | 0 | 0 | 0 | 0 | 0 | 0 | 0 | 0 | 0 | 0 | 0 | 0 | 0 | 0 | 0 | 0 | 0 | 0 | 0 | 0 | 0 | 0 | 0 | 0 | 0 | 0 | 0 | 0 | 0 | 0 | 0 | 0 | 0 | 0 |
| | SpcFG_like | SpcF-/SpcG-like glycosyltransferase | 0 | 0 | 0 | 0 | 0 | 0 | 0 | 0 | 0 | 0 | 0 | 0 | 0 | 0 | 0 | 0 | 0 | 0 | 0 | 0 | 0 | 0 | 0 | 0 | 0 | 0 | 0 | 0 | 0 | 0 | 0 | 0 | 0 | 0 | 0 | 0 | 0 | 0 |

**Supplementary Table S4** Metatranscriptome datasets that were analysed. The datasets contain single (S) or paired end (PE) reads. The No. of reads column counts both forward and reverse reads. NA = not applicable. Human infant metatranscriptome data were deposited on the MG-RAST server.

| *Ecological niche* | *Individual* | *Sample accession/Bioproject* | *Read type* | *No. of reads* | *Mean read length* | *No. of non-rRNA reads* |
|---|---|---|---|---|---|---|
| Human (adult) microbiota (ileostomy effluent) | 1 | SRP020487 | S | 31,180,479 | 101 | 1,250,953 |
| | 2 | SRP020487 | S | 29,709,297 | 101 | 6,089,426 |
| | 3 | SRP020487 | S | 21,557,933 | 101 | 2,479,568 |
| | 4 | SRP020487 | PE | 84,423,774 | 101 | 16,605,439 |
| Human (infant) microbiota (fecal samples) | 1 (2B) | 4621794.3 and 4621795.3 | PE | 3,182,430 | 147 | 42,646 |
| | 1 (2D_1) | 4621796.3 and 4621797.3 | PE | 3,420,018 | 145 | 53,053 |
| | 1 (2D_2) | 4621798.3 and 4621799.3 | PE | 3,100,870 | 144 | 2,181,059 |
| | 1 (2E) | 4621800.3 and 4621801.3 | PE | 3,973,528 | 143 | 57,690 |
| Mouse microbiota (cecum) | 1 | SRP043409 | S | 34,891,288 | 101 | 2,021,316 |
| | 2 | SRP043409 | S | 32,927,387 | 101 | 4,847,143 |
| | 3 | SRP043409 | S | 31,147,539 | 101 | 3,780,547 |
| | 4 | SRP043409 | S | 19,297,845 | 101 | 1,909,103 |
| | 5 | SRP043409 | S | 18,142,320 | 101 | 2,193,310 |
| | 6 | SRP043409 | S | 14,021,215 | 101 | 2,052,744 |
| | 7 | SRP043409 | S | 16,059,372 | 101 | 3,070,674 |
| | 8 | SRP043409 | S | 24,949,630 | 101 | 3,662,649 |
| | 9 | SRP043409 | S | 177,005 ,414 | 101 | 25,865,723 |
| | 10 | SRP043409 | S | 13,448,115 | 101 | 1,386,084 |
| | 11 | SRP043409 | S | 18,255,917 | 101 | 4,072,135 |
| | 12 | SRP043409 | S | 23,463,159 | 101 | 4,255,273 |
| | 13 | SRP043409 | S | 32,948,533 | 101 | 5,702,133 |
| | 14 | SRP043409 | S | 24,004,936 | 101 | 1,786,755 |
| | 15 | SRP043409 | S | 17,712,676 | 101 | 1,528,048 |
| Pig microbiota | 1 | ERS569568 | S | 25,819,747 | 101 | 6,089,501 |
| (proximal colon) | 2 | ERS569569 | S | 20,249,846 | 101 | 2,627,182 |
| | 3 | ERS569570 | S | 21,370,543 | 101 | 2,027,873 |
| | 4 | ERS569571 | S | 24,153,712 | 101 | 2,145,670 |
| | 5 | ERS569572 | S | 38,727,560 | 101 | 4,009,914 |
| | 6 | ERS569573 | S | 21,710,716 | 101 | 3,187,697 |
| Sea | NA | CAM_PROJ_Sapelo2008 | | 2,181,899 | 210 | 1,128,161 |

| | | | | | | |
|---|---|---|---|---|---|---|
| bacterioplankton* | | | S | | | |
| Sponge (*Crambe crambe*) | NA | ERS566232 | PE | 222,545,936 | 101 | 183,623,826 |
| Sea floor (5 m depth)** | NA | SRR571458 | S | 89,774,847 | 204 | 86,127,866 |
| Sea floor (30 m depth)** | NA | SRR948190 | S | 112,049,971 | 204 | 106,253,810 |
| Sea floor (50 m depth)** | NA | SRR948292 | S | 113,798,353 | 204 | 77,710,334 |
| Sea floor (70 m depth)** | NA | SRR948285 | S | 161,875,076 | 204 | 134,860,358 |
| Sea floor (91 m depth)*** | NA | SRR948295 | S | 170,339,305 | 204 | 128,240,508 |
| Sea floor (159 depth)** | NA | SRR948297 | S | 126,247,250 | 204 | 105,631,512 |
| Forest soil*** | NA | SRR091238 | S | 1,188,352 | 385 | 1,073,972 |


* Gifford SM, Sharma S, Rinta-Kanto JM, Moran MA (2011). Quantitative analysis of a deeply sequenced marine microbial metatranscriptome. *ISME J* **5:** 461-472.

** Orsi WD, Edgcomb VP, Christman GD, Biddle JF (2013). Gene expression in the deep biosphere. *Nature* **499:** 205-208.

*** Stewart FJ, Sharma AK, Bryant JA, Eppley JM, DeLong EF (2011). Community transcriptomics reveals universal patterns of protein sequence conservation in natural microbial communities. *Genome Biol* **12:** R26.
